# Family socioeconomic status and nutrition habits of 7–8 year old children: cross-sectional Lithuanian COSI study

**DOI:** 10.1186/s13052-015-0139-1

**Published:** 2015-04-23

**Authors:** Aušra Petrauskienė, Vilma Žaltauskė, Edita Albavičiūtė

**Affiliations:** Institute of Health Research, Academy of Medicine, Lithuanian University of Health Sciences, Kaunas, Lithuania

**Keywords:** Children, Nutrition habits, Socioeconomic status

## Abstract

**Background:**

Nutritional habits are a useful way to characterize whole diets and they are also known to be influenced by a wide range of social and economic factors. The above factors in each country may have different effect on children’s eating habits. In Lithuania the data of children nutrition in association with socio-economic status of family is poor. There are few studies done, where links between nutrition habits of children and socio-economic status of family was evaluated. The aim of this paper is to evaluate association among nutrition habits of first-formers and family socio-economic status in Lithuania.

**Methods:**

Data were obtained participating in the international study, which was performed in all ten districts of Lithuania. A cross-sectional study was carried out in 2010, using the protocol and methodology prepared by the experts from the WHO and countries participating in the Initiative. The data were collected by means of COSI standardized questionnaire, which was filled out by parents of selected first-formers’. In this paper a part of questions regarding children nutrition habits and parents’ socio-economic status is presented. Statistical analysis was performed by using SPSS 20.0 software for Windows. Correlation among variables was evaluated by *χ*^2^. Links among nutrition habits of first-formers and family socioeconomic status were determined using binary logistic regression to calculate odds ratios (OR) and 95% confidence intervals (CI). For all tests p < 0.05 was considered significant.

**Results:**

It was established that the majority (76%) of Lithuanian first-formers eat breakfast every day or 4–6 times a week. Significant differences were found between breakfast consumption and gender – girls eat breakfast less frequently than boys. Odds ratio of children daily breakfast consumption were 1.3 times higher in families where fathers’ were older than 30 years comparing with younger fathers. Meanwhile mothers’ age had significant influence just on children daily soft drinks with sugar consumption.

**Conclusions:**

Results from the national survey of primary school age children of Lithuania reveals that family socio-economic position plays one of the major role in breakfast, fresh fruit and soft drinks with sugar consumption among younger school age children.

## Introduction

Healthy diet of children is associated with positive affect on children growth, development of musculoskeletal, cardiovascular and mental health [1-3]. Nutritional habits are a useful way to characterize whole diets and they are also known to be influenced by a wide range of social and economic factors [4-8]. The above factors in each country may have different effect on children’s eating habits. Some of the research show that children and adolescents from low income families tend to consume less fruit and vegetables and more sugar, fats, processed meat, soft drinks, salty snacks compared with those from higher social class households [9-15]. According to researchers the causal mechanisms may be that healthy foods are frequently more expensive, families in poverty live options or families on lower incomes differ in terms of their education and food culture, leading them to make less healthy food choices in areas with lower availability of healthy food [9,15]. Therefore understanding these factors is of great importance for the development of relevant new health policies, programmes, and interventions. A new WHO (World Health Organization) policy for health – Health 2020: the European Policy for Health and Well-being is seeking to stop growing social and health inequalities in Europe. One of strategic goals of Lithuania participating in the policy is to develop healthy eating habits [16]. In Lithuania the data of children nutrition in association with socio-economic status of family is poor. There are few studies done, where links between nutrition habits of children and socio-economic status of family was evaluated [17]. There is a need to explore and better understand the key determinants of children nutritional habits within a socio-economic frame-work in Lithuania. The aim of this paper is to evaluate association among nutrition habits of first-formers and family socio-economic status in Lithuania.

## Material and methods

WHO Regional Office for Europe established a European childhood obesity surveillance system (COSI). Data were obtained participating in the international study, which was performed in all ten districts of Lithuania. A cross-sectional study was carried out in 2010, using the protocol and methodology prepared by the experts from the WHO and countries participating in the Initiative. A multilevel sampling method (district, school and class) was employed for composing a nationally representative sample (6). The sample size in each of all ten districts of the country was calculated following the data from the Department of Statistics of Lithuania about the number of targeted children. Schools were randomly selected from the list of Ministry of Education and Science. In the 57 municipalities 155 schools were selected for the survey. First class was chosen as a sampling unit.

### Questionnaire

The data were collected by means of COSI standardised questionnaire, which was filled out by parents of selected first-formers’. The optional items of the questionnaire involved the information on children lifestyle and their families’ characteristics. In this paper a part of questions regarding children nutrition habits and parents’ socio-economic status is presented. The questions concerning habitual food consumption has not been validated for estimating total intakes of energy or nutrients but is appropriated for exploring dietary patterns on the basis of frequencies. The alternative frequencies for food and drink items and meal patterns were ‘never’, ‘once or twice per month’, ‘1 – 3 times a week’, ‘4 – 6 times a week’, and ‘every day’. Socio-economic characteristics of family were evaluated by parent’s age, education, occupation, income, and marital status (Table 1). The questionnaires were completed by 4517 parents of first-formers, response rate – 73.2%.

**Table 1:** **Frequencies of first-formers food consumption**

	**Children age**		**Gender**		**Total % (n)**
	**7**	**8**	**Boys**	**Girls**	
	**% (n)**	**% (n)**	**% (n)**	**% (n)**	
**Breakfast consumption**					
Every day and 4–6 times a week	76.2(2386)	75.4(1020)	77.6(1713)	74.4(1693)	76(3406)
1-3 times a week and never	23.8(746)	24.6(332)	22.4(495)	25.6(583)	24(1078)
**Total**	100 (3132)	100(1352)	100 (2208)	100 (2276)	100 (4484)
***χ*** 2 P*	-		0.012		
**Fresh fruits consumption**					
Every day and 4–6 times a week	62.9(1934)	57.5(762)	59.9(1303)	62.6(1393)	61.3(2696)
1-3 times a week and never	37.1(1140)	42.5(564)	40.1(872)	37.4(832)	38.7(1704)
**Total**	100 (3102)	100(1341)	100 (2275)	100 (2225)	100 (4400)
***χ*** 2 P*	0.001		-		
**Fresh vegetables consumption**					
Every day and 4–6 times a week	40.3(1183)	40.8(525)	39.7(822)	41.1(886)	40.4(1708)
1-3 times a week and never	59.7(1755)	59.2(763)	60.3(1247)	58.9(1271)	59.6(2518)
**Total**	100 (2938)	100 (1288)	100 (2069)	100 (2157)	100 (4226)
***χ*** 2 P*	-		-		
**Soft drinks with sugar consumption**					
Every day and 4–6 times a week	15.9(373)	17.2(178)	16.7(285)	15.9(266)	16.3 (551)
1-3 times a week and never	84.1(1976)	82.8(857)	83.3(1423)	84.1(1410)	83.7(2833)
**Total**	100 (2349)	100 (1035)	100 (1708)	100 (1676)	100 (3384)
***χ*** 2 P*	-		-		
**French fries, burgers, pizzas consumption**					
Every day and 4–6 times a week	5(130)	6.1(67)	5.5(104)	5(93)	5.3(197)
1-3 times a week and never	95(2488)	93.9(1040)	94.5(1773)	95(1755)	94.7(3528)
**Total**	100 (2618)	100 (1107)	100 (1877)	100 (1848)	100 (3725)
***χ*** 2 P*	-		-		

P* - significance comparing groups (Chi-squares test).

### Statistical analysis

Statistical analysis was performed by using SPSS 20.0 software for Windows. Descriptive statistics was applied. Children’s nutrition habits were analyzed and compared according to their age and sex. Correlation among variables was evaluated by *χ*^2^. Links among nutrition habits of first-formers and family socioeconomic status were determined using binary logistic regression to calculate odds ratios (OR) and 95% confidence intervals (CI). For all tests p < 0.05 was considered significant.

### Variables

#### Dependent variables

Parents were asked to indicate the frequency of their children eating breakfast, fresh fruit and vegetables, soft-drinks with sugar consumption by ticking one of the following five responses as it was mentioned above. Response options were recoded into dichotomous outcome variables (1 = daily; 0 = less then daily). French fries, burgers, pizzas consumption were also recoded into dichotomous outcome variables (1 = daily or 4–6 times a week; 0 = less then 4 times a week).

#### Independent variables

Five socio-economic status variables (parent’s age, marital status, education, occupation and income) were used in the analyses. Parental age was recoded into three categories (younger than 29 years; from 30 to 39; 40 year and older). Parental occupation from five possible response options was recoded into three categories (low – completed high school; medium – completed college; high – completed graduate degree). Parental occupation from eight response options was recoded into three categories (work in state institution; work in non-state institution; unemployed). Family income from five response option was recoded into three categories (low – 400–600 Lt; middle – 600–1200 Lt and high >1200 Lt per month), later during binary logistic regression calculation family income from three option was recoded into two categories (low <1200 Lt and high >1200 Lt) as middle income were merged with low income group.

## Results

It was established that the majority (76%) of Lithuanian first-formers eat breakfast every day or 4–6 times a week. Significant differences were found between breakfast consumption and gender – girls eat breakfast less frequently than boys. Fresh fruit and vegetable consumption is very important determinant regarding healthy lifestyle. While analyzing the data significant differences were found among first-formers age and fresh fruit consumption: higher percent of 7 year old children consumed fruits every day in comparison with 8 year olds. No differences were observed among gender of respondents and fresh fruit as well as vegetable consumption (Table 1). Unhealthy diet products (soft-drinks with sugar, French fries, burgers, pizzas) were consumed rarely: most of first-formers (from 82.8% to 95%) eat these products 1–3 times a week or never and no significant differences were observed between gender and age.

The analysis of family socio-economic factors revealed that significant differences were found between parental age and their education, occupation, family income and marital status. Almost half of parents (45.3% of fathers and 46.7% of mothers respectively) reported about completed high school and one-third (35.8% of fathers and 34.4% of mothers respectively) of parents had completed graduated degree. Low educated fathers and mothers were mainly younger than 29 years old (65.5% and 68.1% respectively). Generally 73.2% of fathers and 66.8% of mothers were employed, males – more frequently in private sector, females – in state institutions. Analysing by age it was determined that unemployment was more frequent in the group of the youngest parents, especially among women. More than half of respondents indicated low, one third – average and one in ten – high family income. The majority of children lived in two parent family (Table 2).

**Table 2:** **Socio-economic characteristics of families**

**Parents’ age**							**Gender**	
	**Mothers**			**Fathers**				
	**<29 yr.**	**30-39 yr.**	**40 yr. > more % (n)**	**<29 yr.**	**30-39 yr.**	**40 yr. > more % (n)**	**Males**	**Females**
	**% (n)**	**% (n)**		**% (n)**	**% (n)**		**% (n)**	**% (n)**
**Education**								
Low (completed high school)	68.1 (584)	43.5 (1158)	35.2 (298)	65.5 (205)	45.1 (1169)	45.3 (1857)	45.3(1857)	46.7 (2041)
Medium (completed college)	10.1 (87)	18.7 (497)	28.3 (239)	11.5 (36)	17.9 (464)	23.00 (275)	18.9 (775)	18.8 (823)
High (completed graduate degree)	21.8 (187)	37.8 (1007)	36.5 (309)	23.00 (72)	37.00 (960)	36.5 (436)	35.8 (1468)	34.4 (1503)
**Total**	100 (858)	100 (2663)	100 (846)	100 (313)	100 (2593)	100 (1194)	100 (4100)	100 (4367)
***χ*** **2 value**	*χ*2 = 237.389; df = 4; p < 0.001			*χ*2 = 71.211; df = 4; p < 0.001				
**Family income**								
Low (400–600 Lt)	66.00 (538)	55.1 (1401)	56.7 (459)	69.8 (210)	54.9 (1367)	55.8 (632)	57.6 (2398)	56.3 (2209)
Middle (600–1200 Lt)	27.5 (224)	31.4 (798)	33.6 (272)	25.2 (76)	32.2 (802)	32.9 (372)	31.1 (1294)	39.2 (1746)
High (>1200 Lt)	6.5 (53)	13.5 (342)	9.8 (79)	5.00 (15)	12.8 (319)	11.3 (128)	11.4 (474)	11.8 (462)
**Total**	100 (815)	100 (2541)	100 (810)	100 (301)	100 (2566)	100 (1132)	100 (4166)	100 (3921)
***χ*** **2 value**	*χ*2 = 46.506; df = 4; p < 0.001			*χ*2 = 29.444; df = 4; p < 0.001				
**Occupation**								
Work in state institutions	13.9 (122)	29.3 (796)	36.3 (311)	9.1 (29)	18.3(487)	22.6 (274)	18.9 (790)	27.6 (1229)
Work in private sector	39.7 (349)	40.8 (1109)	33.6 (288)	50.9 (163)	57.3 (1523)	48.4 (587)	54.3 (2273)	39.2 (1746)
Unemployed	46.4 (408)	29.9 (814)	30.00 (257)	40.00 (129)	24.3 (646)	29.00(352)	26.8 (1126)	33.2 (1479)
**Total**	100 (581)	100 (2719)	100 (856)	100 (320)	100 (2656)	100 (1213)	100 (4189)	100 (4454)
***χ*** **2 value**	*χ*2 = 125.486; df = 4; p < 0.001			*χ*2 = 67.634; df = 4; p < 0.001				
**Marital status**								
Married							84.8 (3492)	80.3 (3505)
Single, divorced, other							15.2 (627)	19.7 (860)
**Total**							100 (4119)	100 (4365)
***χ*** **2 value**								

P* - significance comparing groups (Chi-squares test).

Logistic regression analysis revealed that mothers’ and fathers’ age had different influence on children habitual food consumption. Odds ratio of children daily breakfast consumption were 1.3 times higher in families where fathers’ were older than 30 years comparing with younger fathers. Meanwhile mothers’ age had significant influence just on children daily soft drinks with sugar consumption. Odds ratio of daily soft drinks with sugar consumption was 1.22 times smaller in families where mothers’ were older than 30 years comparing with younger ones. The analysis established that mothers’ age had no significant impact for children breakfast and fresh fruit consumption, contrary to parental education. It was determined that odds ratio of every day fresh fruit consumption was 1.3 and 1.4 higher of those children whose mothers and fathers had completed graduate degree comparing with low educated parents. Also it was determined that odds ratio of children every day fresh fruit consumption 1.3 and 1.4 higher of those children whose mothers and fathers had completed graduate degree comparing with low educated parents. High parental education had influence on first-formers daily soft drinks with sugar consumption. Odds ratio of soft drinks with sugar consumption was 2.6 and 1.7 times lower among first-formers whose mothers and fathers had high education compared with low educated parents (Table 3). Logistic regression analysis established that family income and marital status had significant impact for children’s daily fruit and soft drinks with sugar consumption, but no impact for daily breakfast consumption. Odds ratio of children fresh fruit consumption was 1.5 times higher in families with high income comparing with low income families. Odds ratio of first-formers soft beverage with sugar consumption was 1.6 and 1.3 times lower in full or high income families compared with single parent, low income families (Table 4).

**Table 3:** **Odds Ratios of children daily breakfast, fresh fruit and soft drinks with sugar consumption by parents’ age and education**

	**Daily breakfast consumption**		**Daily fresh fruit consumption**		**Daily soft drinks with sugar consumption**	
	**Proportion %**	**OR (95% CI)**	**Proportion %**	**OR (95% CI)**	**Proportion %**	**OR (95% CI)**
**Age females (mothers)**						
<29 yr.	70.4%	1	56.2%	1	21.7%	1
30-39 yr.	76.6%	1.135 (0.974; 1.323)	61.1%	1.097 (0.955; 1.259)	14.7%	*0.823 (0.689; 0.984)*
40 yr. > more	77.4%	1.122 (0;898 1.402**)**	61.8%	1.051 (0.863; 1.280)	13.4%	0.817 (0.624; 1.070)
**Age males (fathers)**						
<29 yr.	68.7%	1	55.1%	1	21.6%	1
30-39 yr.	75.8%	*1.267 (1.027; 1.564)*	60.5%	1.071 (0.88; 1.303)	15.3%	0.878 (0.688; 1.120)
40 yr. > more	77.3%	*1.291 (1.004; 1.66* ***1)***	62.0%	1.112 (0.884; 1.400)	13.8%	0.864 (0.641; 1.163)
**Education fathers**						
Low (completed high school)	73.1%	1	57.1%	1	19.2%	1
Medium (completed college)	76.2%	1.066 (0.927; 1.266)	61.6%	1.097 (0.97; 1.241)	13.7%	0.892 (0.752; 1.058)
High (completed graduate degree)	82.6%	*1.440 (1.220; 1.700)*	69.1%	*1.369 (1.189; 1.576)*	7.2%	*0.581 (0.461; 0.731)*
**Education mothers**						
Low (completed high school)	72.0%	1	55.5%	1	22.6%	1
Medium (completed college)	76.3%	1.130 (0.976; 1.309)	61.9%	*1.170 (1.028; 1.331)*	13.4%	*0.616 (0.516; 0.737)*
High (completed graduate degree)	80.5%	*1.293 (1.119; 1.492)*	66.7%	*1.338 (1.224; 1.573)*	7.9%	*0.392 (0.229; 0.669)*

**Table 4:** **Odds Ratios of children daily breakfast, fresh fruit and soft drinks with sugar consumption by family income and marital status**

	**Daily breakfast consumption**		**Daily fresh fruit consumption**		**Daily soft drinks with sugar consumption**	
	**Proportion %**	**OR (95% CI)**	**Proportion %**	**OR (95% CI)**	**Proportion %**	**OR (95% CI)**
**Family income**						
Low (<1200 Lt)	75.0%	1	58.6%	1	16.8%	1
High (>1200 Lt)	77.9%	1.142 (0.978; 1.334)	68.7%	*1.547 (1.346; 1.779* **)**	11.0%	*0.636 (0.518; 0.780* ***)***
**Marital status**						
Divorced	73.7%	1	57.3%	1	19.5%	1
Married	75.9%	1.110 (0.979; 1.259)	60.90%	1.086 (0.970; 1.216)		*0.772 (0.668; 0.892)*

## Discussion

Breakfast consumption is very important for children future health [18-22]. Epidemiological research claims that despite the potential importance of breakfast consumption, the prevalence rates of breakfast skipping among children and adolescents has increased in the past few decades [18,22,23]. Current study findings indicate that 76% of Lithuanian first-formers eat breakfast every day, girls less frequently than boys. Our national data results exhibit lower prevalence of daily breakfast than the average rates of the same COSI study of Portuguese primary school age children. It is reported that in 2010 95.1% of 6–8 year old Portuguese children have breakfast every day [24]. But our results about having breakfast every day were significantly better than the results of Western Sicilia primary school children. The study conducted in Italy when 8–11 year old children were examined, revealed that only 54% of respondents have breakfast every day [25]. Comparing our data with the results of other national survey in Lithuania, primary school-age children eat breakfast more frequently than 11-year olds from Health Behaviour in School-Aged Children (HBSC) study performed in 2010, which results show that 65% of teenagers eat breakfast every [26]. The differences between the results of these two national studies’ can probably be explained by stronger parental attention and influence for first-formers eating patterns and different age of respondents. Many international studies confirm that the prevalence of regular breakfast consumption tends to decrease as children grow older [18,27,28]. Another important children’s eating behaviour component is consuming a diet high in fruits and vegetables which is associated with lower risks of numerous chronic diseases. According to our study results daily intake of fresh fruit and vegetable is 61.3% and 40.4% respectively. Our study results on children fresh fruit and vegetable consumption rates differ from HBSC (2010) study. Daily intake of fresh fruit is twofold lower by 11 year olds (30%) but daily vegetable consumption is 1.4 times higher (54.5%) [26]. Such differences can be explained by the fact that primary school age children more often eat at home, they prefer to eat fruits more than vegetables, as fruits are usually sweet, juicy, and can be eaten fresh, and adolescents are more influenced by friends, social advertisements and willingness to try new snacks, drinks, instead of eating fruits, but perhaps vegetable salads as snacks are also more popular among older children.

Parents play a major role in the development of healthy eating habits of their children through a variety of mechanisms including a healthy diet, the availability and accessibility of nutritious foods at home, and the development of attitudes, values, preferences and role modelling [22-24]. The main findings of this study confirm links between first-formers nutrition habits and family socio-economic status in Lithuania. Parent’s income is one of the primary indicators of socio-economic status. Our results show that parent’s higher education and income have significant positive impact on daily breakfast, fresh fruit consumption of first-formers compared with lower socio-economic position families. In other studies family income is also mentioned as factor linked with higher intake of fruit and vegetables among children [22,23]. The similar outcome presents HBSC study. In the international report on social determinants of health and well-being among young people it is stated that adolescents from high-affluence families in most countries and regions were significantly more likely to report eating breakfast, fresh fruit daily [26]. Current study findings indicate that odds ratio of children soft drinks with sugar consumption were lower in families with high income comparing with low income families. Many studies from different countries confirm these links and claim that prevalence of children and adolescents’ unhealthy behavior including diet tend to be higher in families with low socio-economic position compared with high social class households [19,29-35]. Interesting results of soft-drinks consumption are presented by CA Vereecken at all. It is established that soft drinks consumption is lower among pupils of higher parental occupational status in Northern, Southern and Western European countries, but not in Central and Eastern European countries. Analysing the HBSC 2001/2002 survey results the authors stated that only in Central and Eastern European countries is observed significant increase in soft drink consumption with increasing family affluence [36]. We can state that Lithuanian situation has changed during more than ten year period and there are no differences from Western European countries.

### Strengths and limitations

Among the strengths of the present study this is the first study to assess first-formers nutritional habits and 4 socio-economic aspects of family (education, occupation, family income and marital status. The present study has some limitations. Firstly this study has a cross-sectional design, which does not allow to draw conclusions about causal relationships. Secondly, assessments of child’s habitual food consumption were based on parent’s self-reports which cannot exclude misreporting, recall biases.

## Conclusions

Results from the national survey of primary school age children of Lithuania reveals that family socio-economic position plays one of the major role in breakfast, fresh fruit and soft drinks with sugar consumption among younger school age children. Likelihood of daily breakfast, and fresh fruit consumption of first-formers were higher in high socio-economic status families compared with low. Opposite results were found in soft drinks with sugar consumption. This social patterning should be recognized in public health interventions.
